# Influenza vaccine effectiveness in Europe and the birth cohort effect against influenza A(H1N1)pdm09: VEBIS primary care multicentre study, 2023/24

**DOI:** 10.2807/1560-7917.ES.2025.30.23.2500011

**Published:** 2025-06-12

**Authors:** Esther Kissling, Marine Maurel, Francisco Pozo, Gloria Pérez-Gimeno, Silke Buda, Noémie Sève, Lisa Domegan, Mariëtte Hooiveld, Beatrix Oroszi, Iván Martínez-Baz, Raquel Guiomar, Neus Latorre-Margalef, Ivan Mlinarić, Mihaela Lazar, Jaume Giménez Duran, Ralf Dürrwald, Vincent Enouf, Adele McKenna, Marit de Lange, Gergő Túri, Camino Trobajo-Sanmartín, Verónica Gomez, Tove Samuelsson Hagey, Vesna Višekruna Vučina, Maria Carmen Cherciu, Miriam García Vazquez, Annika Erdwiens, Shirley Masse, Charlene Bennett, Adam Meijer, Katalin Kristóf, Jesús Castilla, Ana Paula Rodrigues, Sanja Kurečić Filipović, Alina Elena Ivanciuc, Sabrina Bacci, Marlena Kaczmarek, on behalf of the European primary care VE group

**Affiliations:** 1 Epiconcept, Paris, France; 2 University of Antwerp, Antwerp, Belgium; 3 National Centre for Microbiology, National Influenza Reference Laboratory, WHO-National Influenza Centre, Institute of Health Carlos III, Madrid, Spain; 4 CIBER de Epidemiología y Salud Pública (CIBERESP), Institute of Health Carlos III, Madrid, Spain; 5 National Centre of Epidemiology, Institute of Health Carlos III, Madrid, Spain; 6 Robert Koch Institute, Berlin, Germany; 7 Sorbonne Université, INSERM, Institut Pierre Louis d'épidémiologie et de Santé Publique (IPLESP), F75012, Paris, France; 8 Health Service Executive-Health Protection Surveillance Centre, Dublin, Ireland; 9 Nivel, Utrecht, the Netherlands; 10 National Laboratory for Health Security, Epidemiology and Surveillance Centre, Semmelweis University, Budapest, Hungary; 11 Instituto de Salud Pública de Navarra – IdiSNA, Pamplona, Spain; 12 Laboratório Nacional Referência Gripe e outros Vírus Respiratórios, Instituto Nacional de Saúde Doutor Ricardo Jorge, Lisbon, Portugal; 13 Department of Microbiology, Public Health Agency of Sweden, Stockholm, Sweden; 14 Division for Epidemiology of Communicable Diseases, Croatian Institute of Public Health, Zagreb, Croatia; 15 National Influenza Centre, Cantacuzino National Military-Medical Institute for Research and Development, Bucharest, Romania; 16 Servicio de Epidemiologia DG Salut Pública, IDISBA (Instituto de Investigaciones Sanitarias de Baleares), Illes Balears, Spain; 17 Centre National de Référence Virus des Infections Respiratoire (CNR VIR), M3P unit, Institut Pasteur Université Paris Cité, Paris, France; 18 National Institute for Public Health and the Environment, Centre for Infectious Diseases Control, Bilthoven, the Netherlands; 19 Epidemiology Department, Instituto Nacional de Saúde Doutor Ricardo Jorge, Lisbon, Portugal; 20 Sección de Vigilancia Epidemiológica de la Dirección General de Salud Pública, Aragón, Spain; 21 Unite des Virus Emergents (UVE: Aix-Marseille Univ, Universita di Corsica, IRD 190, Inserm 1207, IRBA), Marseille, France; 22 National Virus Reference Laboratory, University College Dublin, Dublin, Ireland; 23 Institute of Laboratory Medicine, Semmelweis University, Budapest, Hungary; 24 European Centre for Disease Prevention and Control, Stockholm, Sweden; 25 The members of the European primary care VE group are acknowledged at the end of the article; * These authors contributed equally to this work and share first authorship.

**Keywords:** Influenza, influenza vaccines, vaccine effectiveness, multicentre study, case-control study, imprinting

## Abstract

**Introduction:**

Influenza A(H1N1)pdm09, A(H3N2) and B/Victoria viruses circulated in Europe in 2023/24, with A(H1N1)pdm09 dominance. First influenza infections in childhood may lead to different vaccine effectiveness (VE) in subsequent years.

**Aim:**

The VEBIS primary care network estimated influenza VE in Europe using a multicentre test‐negative study.

**Methods:**

Primary care practitioners collected information and specimens from patients consulting with acute respiratory infection. We estimated VE against influenza (sub)type and clade, by age group and by year of age for A(H1N1)pdm09, using logistic regression.

**Results:**

We included 29,958 patients, with 3,054, 1,053 and 311 influenza A(H1N1)pdm09, A(H3N2) and B cases, respectively. All-age VE against influenza A(H1N1)pdm09 was 52% (95% CI: 44–59). By year of age, VE was 27% (95% CI: −2 to 47) at 44 years with peaks at 72% (95% CI: 52–84) and 54% (95% CI: 41–64) among children and those 65 years and older, respectively. All-age A(H1N1)pdm09 VE against clade 5a.2a was 41% (95% CI: 24–54) and −11% (95% CI: −69 to 26) against clade 5a.2a.1. The A(H3N2) VE was 35% (95% CI: 20–48) among all ages and ranged between 34% and 40% by age group. All-age VE against clade 2a.3a.1 was 38% (95% CI: 1–62). All-age VE against B/Victoria was 83% (95% CI: 65–94), ranging between 70 and 92% by age group.

**Discussion:**

The 2023/24 VEBIS primary care VE against medically attended symptomatic influenza infection was high against influenza B/Victoria, but lower against influenza A(H1N1)pdm09 and A(H3N2). Clade- and age-specific effects may have played a role in the lower A(H1N1)pdm09 VE.

**Key public health message**

**What did you want to address in this study and why?**

Influenza viruses come in different (sub)types and evolve rapidly. Effectiveness of the annual influenza vaccine can vary year to year. A person’s first influenza infection in childhood may influence vaccine effectiveness (VE) later in life. We estimated effectiveness of the 2023/24 influenza vaccine in patients consulting a doctor for acute respiratory infection. We explored how first influenza infection may have affected VE against one subtype: A(H1N1)pdm09.

**What have we learnt from this study?**

The study found that influenza vaccination prevented half of all influenza infections among vaccinated people of any age. This protection varied by influenza (sub)type, with the vaccine preventing between around a third and four-fifths of infections, depending on (sub)type. The vaccine’s effect varied by age group for subtype A(H1N1)pdm09: it was more effective among children and older adults and provided lower protection among middle-aged adults.

**What are the implications of your findings for public health?**

We noted differences by age group, with lower VE among those people whose first infection in life can be assumed to have been an A(H1N1) influenza infection with virus strains circulating in 1976–1984. More research on how first influenza infection shapes the current influenza VE is needed. While the vaccine’s effect can vary based on specific factors, it remains a key protective measure against disease.

## Introduction

In February 2023, the World Health Organisation (WHO) recommended that the 2023/24 trivalent egg-based influenza vaccine for the northern Hemisphere should include an A/Victoria/4897/2022 (H1N1)pdm09‐like virus, an A/Darwin/9/2021 (H3N2)-like virus and a B/Austria/1359417/2021 (B/Victoria lineage)-like virus. The recommendation for the cell-based vaccine was to include an A/Wisconsin/67/2022 (H1N1)pdm09-like virus, an A/Massachusetts/18/2022 (H3N2)-like virus and the same B virus as for the trivalent egg-based vaccine. Quadrivalent vaccines containing two influenza B virus lineages were recommended to contain additionally the B/Phuket/3073/2013 (B/Yamagata lineage)-like virus [1].

Influenza A circulated in Europe during the 2023/24 influenza season, with a predominance of influenza A(H1N1)pdm09 over influenza A(H3N2), as well as some influenza B/Victoria later in the season [2].

First influenza infections in life may influence the immunity response to subsequent influenza infections and vaccinations, also known as immunological imprinting [3-5]. The influenza viruses of first exposure differ by birth cohort [6] because of antigenic shift and drift. These include major changes in the circulating virus, e.g. a shift from influenza A(H1N1) to A(H2N2) or A(H3N2), or antigenic drift through amino acid substitutions/deletions at key positions in the influenza haemagglutinin [6,7]. Different A(H1N1)pdm09 vaccine effectiveness (VE) by birth cohort potentially related to immunological imprinting has been suggested before [8-10].

The VEBIS (Vaccine Effectiveness, Burden and Impact Studies) network, previously I-MOVE (Influenza Monitoring Vaccine Effectiveness in Europe), has been estimating influenza VE at primary care level in the European Union (EU) and European Economic Area (EEA) since the 2008/09 season. It is a multicentre study including 10 European countries [11-16]. In countries participating in the study, influenza vaccination is recommended for older adults (people ≥ 60 or ≥ 65 years, depending on the country), for people in medical risk groups for severe disease, and for healthy children in some countries, e.g. Ireland (age 2–17 years) and Spain (age 6 months–4 years) [17,18]. Supplementary Table S1 provides details on influenza vaccination campaigns among those study sites participating in the study.

We present the VEBIS end‐of‐season influenza VE estimates of 2023/24 by (sub)type and by clade, for all ages, by age group and by influenza vaccination target group, among patients presenting with an acute respiratory infection at primary care level. We also investigate potential effect of birth cohort on VE against influenza A(H1N1)pdm09, as differences in VE by birth cohort have been reported previously [8,9,19].

## Methods

The VEBIS study is a multicentre test-negative case–control study. Eleven European study sites from 10 countries (Croatia, France, Germany, Hungary, Ireland, the Netherlands, Portugal, Romania, Spain (two sites: one national and one regional) and Sweden) participated in the 2023/24 season. The methods are based on a generic study protocol, adapted at each site [20].

Participating practitioners collected specimens and interviewed all or a systematic sample of patients consulting for acute respiratory infection (ARI) or influenza-like illness (ILI), depending on study site. Supplementary Table S1 outlines the case definition for recruitment for each site. The common variables collected in all study sites were symptoms, date of onset, date of specimen collection, 2023/24 seasonal influenza vaccination status and date, sex, age and presence of chronic conditions. All specimens were RT-PCR tested for influenza virus.

In the pooled analysis, we included patients with a specimen taken fewer than 8 days after symptom onset and meeting the EU ARI or ILI case definition [21]. Patients testing RT-PCR-positive for influenza virus were designated as cases and those testing RT-PCR-negative for any influenza virus as controls.

For each study site, we included patients presenting with ARI/ILI symptoms ≥ 14 days after the start of national influenza vaccination campaigns. Controls were excluded if they presented before the onset week of the first influenza (sub)type-positive case of the relevant analysis.

A patient was considered as vaccinated if they had received an influenza vaccine 14 or more days before symptom onset. Patients vaccinated 1–13 days before symptom onset were excluded. All the others were classified as unvaccinated. Influenza vaccination status was ascertained from electronic medical records by practitioners, obtained through linkage to national vaccine registries or was self-reported.

We excluded any study site from the pooled analysis that had fewer than 10 influenza (sub)type-specific cases or controls and combined individual patient data. We described cases and controls by baseline characteristics. For VE analyses, we used a one-stage model, with study site as a fixed effect. We estimated influenza VE as (1 − (odds of influenza vaccination among cases/odds of influenza vaccination among controls)) × 100. We conducted a complete case analysis and used logistic regression to estimate the OR, including a priori potential confounding factors: age, sex, presence of at least one commonly collected chronic condition (including lung disease, heart disease, immunodeficiency and diabetes) and date of symptom onset. For continuous variables, we used age categorised into narrow groups (empirically shown to reduce residual confounding in our study over the years: 0–1, 2, 3–4, 5–9, 10–19, 20–29, …, 60–69, ≥ 70 years), age in years as a linear term or as a restricted cubic spline (with three, four or five knots) and symptom onset date as a restricted cubic spline (with three, four or five knots). We used the Akaike information criterion (AIC) to select the best functional form of the continuous variables.

We estimated VE against each influenza (sub)type and, where sample size allowed, stratified by age group (0–17, 18–64, ≥ 65 years) and by target group for influenza vaccination, as defined as those groups where vaccination is fully reimbursed (specific age groups as described in Supplementary Table S1 and those with medical risk conditions).

Sites selected all or a random sample of influenza viruses for genetic sequencing. Sequences were uploaded by each site to GISAID and downloaded at the National Influenza Centre, Madrid, Spain for centralised phylogenetic and amino acid substitution analysis in MEGA7 to determine clade distribution. For the clade‐specific VE analysis, we excluded any study site that had fewer than five cases of that influenza clade.

As not all study sites attempted to sequence 100% of viruses across the season, we carried out an analysis taking the sampling fraction into account. The logistic regression model was weighted using the reciprocal of the fraction of being sequenced (sequencing fraction) within each period with different sampling fractions and robust standard errors were used. The sequencing fraction was defined for each study site for each (sub)type for different periods within the season.

For influenza A(H1N1)pdm09 birth cohort-specific descriptive analyses, we calculated numbers of influenza A(H1N1)pdm09 cases and controls by 5-year age groups and influenza vaccination status, overall and by A(H1N1)pdm09 clade. We calculated birth year by subtracting age from the year of season start (2023) and truncated the data at 90 years, to avoid sparse data among older age groups. For birth cohort-specific A(H1N1)pdm09 VE, we grouped birth years according to first likely influenza A infection in Europe at 1 year of age, with recent (from 1977 onwards) A(H1N1) viruses grouped into major antigenic groups, which are detailed in Supplementary Table S2 [22]. This resulted in the age groups: 0–15, 16–25, 26–38, 39–47, 48–56, 57–67 and 68–90 years. As well as an age-stratified analysis, we also estimated A(H1N1)pdm09 VE by birth year/year of age, using an interaction term between vaccination and age in years, modelled as a restricted cubic spline. We estimated models with five, six and seven knots, using locations as specified by Harrell [23] and equally spaced knots. We selected the best-fitting model among fully adjusted models (adjusted for onset date, chronic condition, sex and study site), using the AIC. We also checked the magnitude of regression coefficients and their standard errors for unstable results.

If the number of cases or controls were fewer than 10 times the number of parameters in a model, we used penalised logistic regression (Firth's method) to assess small sample bias. If the VE differed by 10% or more, we assumed there was a small sample bias and did not present the results.

All analyses were performed in R version 4.3.0 (R Project for Statistical Computing) within the RStudio environment. Additional R packages for analysis included logistf for penalised logistic regression, rms for restricted cubic splines and interactionRCS for the VE by age in years.

## Results

**Figure 1:**
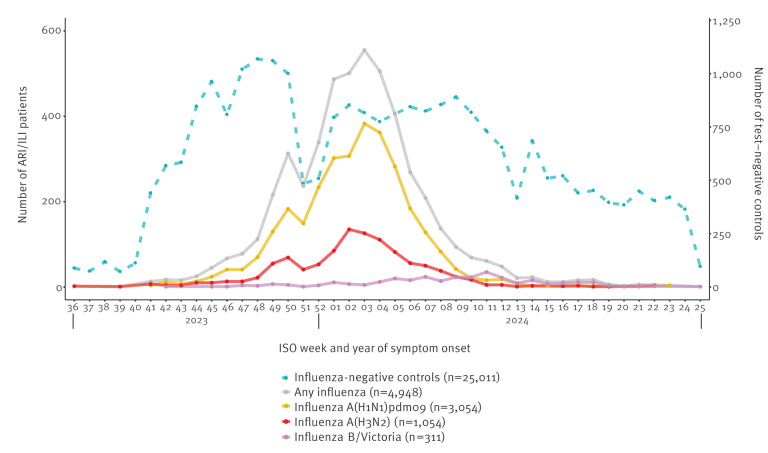
Number of influenza cases and test-negative controls by week of symptom onset, VEBIS primary care study, September 2023–June 2024 (n = 29,959)

ARI: acute respiratory infection; ILI: Influenza-like illness; ISO: International Organization for Standardization.

We included 29,959 eligible patients between week 36 2023 and week 25 2024, of whom 4,948 (17%) were influenza-positive (Figure 1). Among cases, 4,583 had influenza A, 330 influenza B/Victoria and 40 had not typed influenza infections. Among influenza A infections, 3,070 were influenza A(H1N1)pdm09, 1,062 A(H3N2) and 459 unsubtyped influenza A. There were eight influenza A(H3N2) and A(H1N1)pdm09 co-infections and five influenza A(H1N1)pdm09 and B co-infections. For influenza A(H1N1)pdm09, A(H3N2) and B VE analyses, we then excluded two sites (16 patients), one site (eight patients) and seven sites (19 patients), respectively, for small sample size, leaving 3,054, 1,054 and 311 virus infections for analysis. Vaccination campaigns started from week 34 2023, and vaccinations occurred up to week 10 2024 in the study population.

The median age was 37 years among controls and influenza A(H1N1)pdm09 cases, 34 years among influenza A(H3N2) cases and 16 years among influenza B cases. Among controls, 31% were aged 0–17 years compared with 53%, 34%, and 25% among influenza B/Victoria, A(H1N1)pdm09 and A(H3N2) cases, respectively. There were 15% of patients aged 65 years and older among controls, 9% among influenza A(H3N2) cases, 8% among influenza A(H1N1)pdm09 cases and less than 1% among influenza B/Victoria cases (Table 1).

**Table 1:** Characteristics of all influenza, A(H1N1)pdm09, A(H3N2) and B cases and controls VEBIS primary care multicentre study, September 2023–June 2024 (n = 29,959)

Variable			Test-negative controls (n = 25,011)^a^		All influenza cases (n = 4,948)		Influenza A(H3N2) cases (n = 1,054)		Influenza A(H1N1)pdm09 cases (n = 3,054)		Influenza B/Victoria cases (n = 311)	
			n	%	n	%	n	%	n	%	n	%
Median age (IQR) in years			37 (12–56)		36 (12–51)		34 (17–52)		37 (9–52)		16 (9–33)	
**Age group (years) **												
0–17			7,746	31	1,583	32	268	25	1,022	34	165	53
18–64			13,413	54	2,972	60	687	65	1,783	58	144	46
≥ 65			3,852	15	393	8	99	9	249	8	2	1
**Sex**												
Female			14,177	57	2,684	54	587	56	1,627	53	152	49
Male			10,834	43	2,264	46	467	44	1,427	47	159	51
**Seasonal influenza vaccination**												
No			20,244	81	4,456	90	918	87	2,760	90	305	98
Yes			4,767	19	492	10	136	13	294	10	6	2
**Vaccine type used among vaccinated**												
Trivalent vaccine^b^			50	1	4	0	1	1	3	0	0	0
Quadrivalent vaccine egg-passaged		Normal dose, non-adjuvanted, inactivated	2,751	63	298	73	95	81	174	75	4	80
		High dose	341	8	33	8	6	5	26	11	0	0
		Adjuvanted	636	14	30	7	6	5	14	6	0	0
		LAIV	150	3	15	4	6	5	6	3	0	0
Quadrivalent vaccine cell-passaged			477	11	27	7	4	3	8	4	1	20
Missing			362		85		18		63		1	
Median delay between influenza vaccination and onset of symptoms in days (IQR)			95 (51–145)		69 (52–90)		72 (53–90)		69 (51–89)		85 (52–105)	
Median delay between onset of symptoms and swabbing in days (IQR)			3 (2–4)		3 (2–4)		3 (2–4)		2 (2–4)		3 (2–4)	
**Any chronic condition**												
Absence of chronic disease			18,630	74	4,011	81	841	80	2,465	81	285	92
Presence of chronic disease			6,381	26	937	19	213	20	589	19	26	8
**Target group for influenza vaccination^c^**												
No	All		13,059	53	3,165	65	597	58	2,020	67	239	79
	Vaccinated		665	5	117	4	30	5	67	3	6	3
	Unvaccinated		12,394	95	3,048	96	567	95	1,953	97	233	97
Yes	All		11,605	47	1,706	35	429	42	1,001	33	65	21
	Vaccinated		4,055	35	369	22	104	24	224	22	0	0
	Unvaccinated		7,550	65	1,337	78	325	76	777	78	65	100
Missing			347		77		28		33		7	
**SARS-CoV-2 PCR test result**												
Negative			21,987	88	4,807	97	1,025	97	2,972	98	308	99
Positive			2,990	12	131	3	28	3	76	2	3	1
Missing			34		10		1		6		0	

IQR: interquartile range; LAIV: live attenuated influenza vaccine; SARS-CoV-2: severe acute respiratory syndrome coronavirus 2; VEBIS: Vaccine Effectiveness, Burden and Impact Studies.

^a^ Controls for ‘any influenza’ used here (number of controls differs slightly for influenza (sub)types analyses, due to the inclusion criteria)

^b^ All trivalent vaccines were 3Fluart and they were only used in Hungary.

^c^ Target group, varying among countries, included patients with chronic conditions, pregnant women (for some countries), older adults (age ≥ 60 or ≥ 65 years depending on study site), patients belonging to other risk groups (e.g. healthcare workers and other professional groups). In four study sites, it included children (from 6 months or 2-year-olds to 5 or 17 years, depending on study site).

The contribution of cases and controls varied by study site, with Germany contributing 39% of cases among 0–17-year-olds (614/1,583) and Spain contributing 41% (1,224/2,972) and 48% (190/393) cases among 18–64-year-olds and those aged 65 and older, respectively. Further information on numbers of cases and controls by site can be found in Supplementary Table S3.

### Genetic characterisation

Twenty-five percent (258/1,054) of influenza A(H3N2), 37% (1,117/3,054) of influenza A(H1N1)pdm09 and 36% of influenza B viruses (112/311) were sequenced in the study sites and laboratories performing sequencing (Table 2). Among the 258 sequenced influenza A(H3N2) samples, all belonged to clade 2a.3a.1. Among the 1,117 sequenced influenza A(H1N1)pdm09 samples, 874 (78%) belonged to clade 5a.2a and 243 (22%) belonged to clade 5a.2a.1. Among the 112 sequenced influenza B samples, all belonged to clade V1A.3a.2.

**Table 2:** Influenza viruses characterised by clade, VEBIS primary care multicentre study, September 2023–June 2024 (n =1,487)

Characterised viruses	Clade	n	%
Influenza A(H3N2) (n = 258)	2a.3a.1	258	100
Influenza A(H1N1)pdm09 (n = 1,117)	5a.2a	874	78
	5a.2a.1	243	22
Influenza B (n = 112)	V1A.3a.2	112	100

### Influenza vaccination among study participants

Among controls, the proportion vaccinated was 19% (n = 4,767) compared with 10% (n = 492) among influenza cases. Among controls in the target group for influenza vaccination, 35% (n = 4,055) were vaccinated compared with 22% (n = 369) among any influenza cases.

Among vaccinated controls, vaccine type was known for 92% of the patients (n = 4,405/4,767), and most of them (88%; n = 3,878) had received quadrivalent egg‐passaged vaccines. Of the 4,405 controls with available vaccine type information, 2,751 (63%) received a normal dose non‐adjuvanted egg‐passaged inactivated quadrivalent vaccine, 636 (14%) received an adjuvanted vaccine, 341 (8%) received a high dose vaccine, 150 (3%) a live attenuated influenza vaccine, and 477 (11%) a quadrivalent cell‐passaged vaccine.

**Figure 2:**
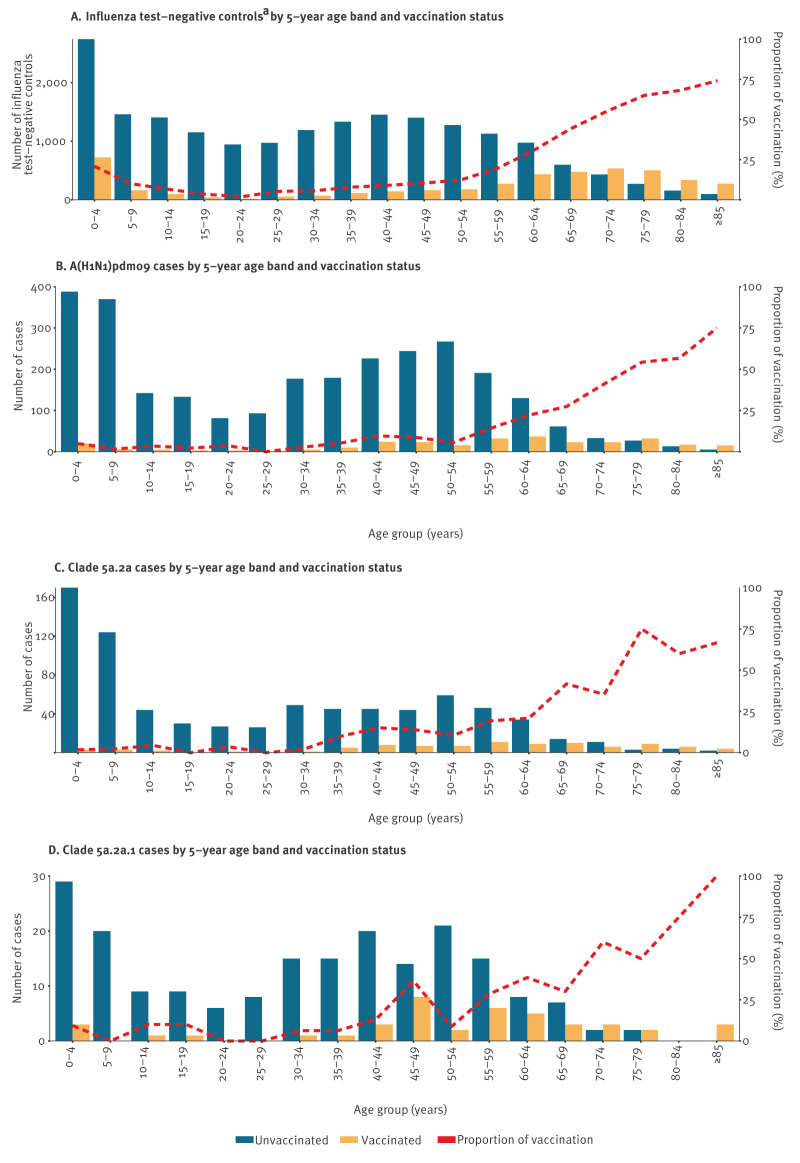
Number of patients by 5-year age band and vaccination status, and proportion of vaccinated cases, for influenza test-negative controls, A(H1N1)pdm09, clade 5a.2a and clade 5a.2a.1 cases, VEBIS primary care study, September 2023–June 2024 (n = 29,959)

^a^ Influenza test-negative controls correspond to the influenza A(H1N1)pdm09 analysis, and controls with onset a week before or after an influenza A(H1N1)pdm09 case in each site were excluded.

Children under 10 years of age and working-age adults were the most affected by influenza A(H1N1)pdm09. The proportion vaccinated among cases increased with age (Figure 2). Among the 45–49-year-olds, the proportion vaccinated was 9% (n = 23/267) among influenza A(H1N1)pdm09 cases, with seven of 51 vaccinated among clade 5a.2a cases and eight of 22 vaccinated among clade 5a.2a.1 cases (Figure 2). Among controls, 10% (n = 163/1,563) were vaccinated in the 45–49-year-old age group. 

Similarly, in the birth cohorts born between 1976 and 1984 (aged 39–47 years), the proportion of vaccinated among cases with clade 5a.2a.1 was 21% (n = 9/43), compared with 8% (n = 38/455) and 12% (n = 12/98) among overall A(H1N1)pdm09 cases and clade 5a.2a cases, respectively. Supplementary Figure S1 provides details on the proportions vaccinated among other birth cohorts.

### Vaccine effectiveness estimates by influenza (sub)type 

Table 3 describes the details of the VE estimates by type and subtype.

**Table 3:** Vaccine effectiveness against any influenza, A(H3N2), A(H1N1)pdm09 and B, overall, by age group, by birth cohort and among the target group for vaccination, VEBIS primary care multicentre case control study, September 2023–June 2024 (n = 29,959)

Study population	n^a^	Cases	Cases vaccinated	Controls	Controls vaccinated	VE^b^	95% CI
Any influenza							
All ages	29,959	4,948	492	25,011	4,767	51	45 to 56
0–17 years	9,329	1,583	64	7,746	1,043	70	61 to 78
18–64 years	16,385	2,972	243	13,413	1,514	41	30 to 49
≥ 65 years	4,245	393	185	3,852	2,210	49	35 to 60
Target group	13,311	1,706	369	11,605	4,055	53	46 to 60
Influenza A(H3N2)							
All ages	24,767	1,054	136	23,713	4,552	35	20 to 48
0–17 years	7,679	268	22	7,411	1,013	40	4 to 64
18–64 years	13,338	687	62	12,651	1,447	34	13 to 52
Target group	11,500	429	104	11,071	3,876	43	27 to 56
Influenza A(H3N2) clade 2a.3a.1							
All ages	13,408	246	28	13,162	2,415	38	1 to 62
18–64 years	6,980	158	10	6,822	696	65	26 to 84
Target group	6,119	90	22	6,029	2,054	56	23 to 75
Influenza A(H1N1)pdm09							
All ages	26,640	3,054	294	23,586	4,611	52	44 to 59
0–17 years	8,358	1,022	34	7,336	1,016	73	61 to 82
18–64 years	14,355	1,783	150	12,572	1,470	40	27 to 51
≥ 65 years	3,927	249	110	3,678	2,125	52	36 to 64
Target group	12,073	1,001	224	11,072	3,925	53	44 to 60
Influenza A(H1N1)pdm09 by birth cohort^c^							
0–15 years	7,803	968	32	6835	996	73	61 to 82
16–25 years	2,304	198	5	2106	61	51	−23 to 84
26–38 years	3,637	406	13	3231	205	63	34 to 81
39–47 years	3,312	455	38	2857	265	16	−25 to 45
48–56 years	3,115	486	41	2629	344	40	13 to 59
57–67 years	3,256	343	67	2913	883	48	30 to 63
68–90 years	3,125	192	93	2933	1795	51	32 to 65
Influenza A(H1N1)pdm09 clade 5a.2a							
All ages	16,624	869	92	15,755	2,958	41	24 to 54
0–17 years	5,604	366	8	5,238	715	76	48 to 88
18–64 years	8,598	434	49	8,164	905	28	−2 to 49
Target group	7,447	248	68	7,199	2,515	49	30 to 63
Influenza A(H1N1)pdm09 clade 5a.2a.1							
All ages	13,051	242	42	12,809	2,303	−11	−69 to 26
18–64 years	6,859	150	26	6,709	707	−36	−126 to 18
Target group	5,926	95	32	5,831	1,955	−11	−89 to 35
Influenza B/Victoria							
All ages	21,511	311	6	21,200	4,326	83	65 to 94
0–17 years	7,008	165	1	6,843	1,031	92	64 to 100
18–64 years	11,196	144	5	11,052	1,337	70	32 to 90

CI: confidence interval; VE: vaccine effectiveness.

^a^ Based on the complete case analysis: records with missing age, sex and chronic condition were dropped.

^b^ Models adjusted by age, sex, presence of at least one of four commonly collected chronic conditions (lung disease, heart disease, immunodeficiency and diabetes), date of symptom onset.

^c^ Corresponding to: 2008–2023 (0–15 years), 1998–2007 (16–25 years), 1985–1977 (26–38 years), 1976–1984 (39–47 years), 1967–1975 (48–56 years), 1956–1966 (57–67 years), 1933–1955 (68–90 years).

#### Any influenza

The all-age VE against any influenza was 51% (95% CI: 45–56). The VE was 70% (95% CI: 61–78) among 0–17-year-olds, 41% (95% CI: 30–49) among 18–64-year-olds and 49% (95% CI: 35–60) among those aged 65 years and older. The VE among patients in the influenza vaccination target group was 53% (95% CI: 46–60) (Table 3); results in graphical format are appended in Supplementary Figure S2.

#### Influenza A(H3N2)

Against influenza A(H3N2), overall VE was 35% (95% CI: 20–48). The VE was 40% (95% CI: 4–64) among 0–17-year-olds and 34% (95% CI: 13–52) among 18–64-year-olds. Sample size did not permit to estimate the VE among those aged 65 years and older. Among patients in the influenza vaccination target group, VE was 43% (95% CI: 27–56) (Table 3); results in graphical format are appended in Supplementary Figure S2.

Among all ages, the VE was 38% (95% CI: 1–62) against clade 2a.3a.1 (Table 3).

#### Influenza A(H1N1)pdm09

The VE against influenza A(H1N1)pdm09 was 52% (95% CI: 44–59). The VE was 73% (95% CI: 61–82) among 0–17-year-olds, 40% (95% CI: 27–51) among 18–64-year-olds and 52% (95% CI: 36–64) among those aged 65 years and older. Among those in the target group for influenza vaccination, VE was 53% (95% CI: 44–60) (Table 3); results in graphical format are appended in Supplementary Figure S2.

The overall VE against clade 5a.2a was 41% (95% CI: 24–54) and −11% (95% CI: −69 to 26) against clade 5a.2a.1 (Table 3).

### Influenza A(H1N1)pdm09 vaccine effectiveness estimates by birth cohort

**Figure 3:**
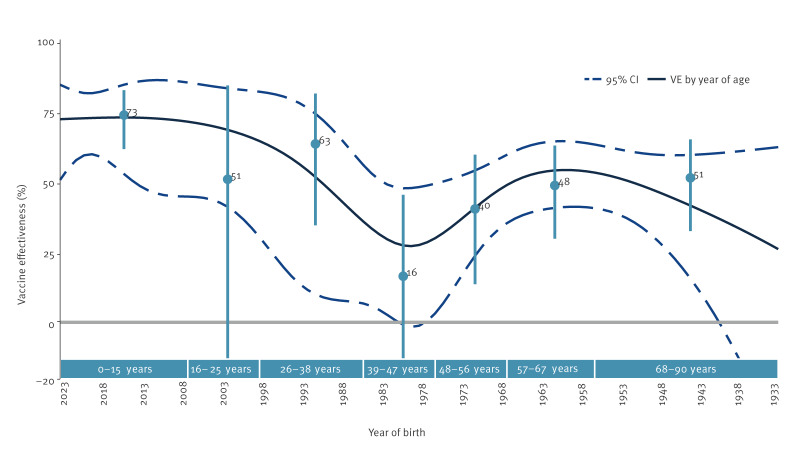
Vaccine effectiveness against influenza A(H1N1)pdm09 by birth cohort / year of age, VEBIS primary care multicentre study, September 2023–June 2024 (n = 29,959)

CI: confidence interval; VE: vaccine effectiveness.

The A(H1N1)pdm09 VE was 73% (95% CI: 61–82), 51% (95% CI: −23 to 84), 63% (95% CI: 34–81), 16% (95% CI: −25 to 45), 40% (95% CI: 13–59), 48% (95% CI: 30–63) and 51% (95% CI: 32–65) among 0–15-year-olds, 16–25-year-olds, 26–38-year-olds, 39–47-year-olds, 48–56-year-olds, 57–67-year-olds and 68–90-year-olds, respectively (Figure 3, Table 3). When modelling VE by age in years (birth year), VE was lowest at age 44 years (birth year 1979) at 27% (95% CI: −2 to 47) (Figure 3). The VE was roughly u-shaped, peaking at age 8 years (birth year 2015) at 72% (95% CI: 52–84) before declining to its lowest point among middle-aged adults and then peaking at age 63 years (birth year 1960) at 54% (95% CI: 41–64).

#### Influenza B/Victoria

The VE against influenza B/Victoria was 83% (95% CI: 65–94). The VE was 92% (95% CI: 64–100) among 0–17-year-olds and 70% (95% CI: 32–90) among 18–64-year-olds. Sample size did not permit to estimate the VE among those aged 65 years and older and among those in the target group for influenza vaccination (Table 3); results in graphical format are appended in Supplementary Figure S2.

## Discussion

In the 2023/24 influenza season, influenza A(H1N1)pdm09 viruses were dominant in the sites included in the VEBIS primary care multicentre study, with influenza A(H3N2) and some B/Victoria co-circulating, reflecting what was reported in Europe [24]. The all-age VE against influenza A(H1N1)pdm09 was 52%. There was indication of birth cohort-specific effects, with VE ranging between 27% and 72% by birth cohort, for those born in 1979 (44 years of age) and 2015 (8 years of age), respectively. The VE was 35% against influenza A(H3N2) among all ages, ranging between 34% and 43% by age and target groups (sample size was too low to estimate VE for those ≥ 65 years). The VE against B was high, at 83% among all ages.

The results were similar to the VEBIS interim 2023/24 estimates against influenza A(H1N1)pdm09 (52% vs 53%, respectively). Higher estimates for the 2023/24 interim A(H1N1)pdm09 VE in primary care settings were found in Canada at 63% (95% CI: 51–72) for all ages and in the United Kingdom (UK) among the 18–64-year-olds at 62% (95% CI: 46–74) [25,26]. However, in the end-of-season United States (US) estimates, VE was lower, at 29% (95% CI: 15–41) among all ages [27]. While our all-age 2023/24 end-of-season VE estimate against influenza A(H1N1)pdm09 was comparable to most estimates from previous seasons in the network since 2009 [10,12-15,28], the 2023/24 VE was lower at 40% among the 18–64-year-olds. Lower VE among working-age adults was also observed in Canada and the US [25,27]. We observed low or no effect of the vaccine against influenza 5a.2a.1, despite the clade match to the vaccine strain. The VE against influenza 5a.2a.1 among 18–64 years was −36% (95% CI: −126 to 18), and the low all-age VE may have been driven by low VE in this age group. The proportion of vaccinated among 5a.2a.1 cases was high at 36% in those aged 45–49 years, compared with 5a.2a cases (14%) and controls (10%). In their 2023/24 interim influenza VE manuscript, Canadian colleagues suggest that an egg-derived mutation in the A(H1N1)pdm09 vaccine high-growth reassortant – the R142K reversion – could play a role in this lower VE against circulating 5a.2a.1 [25]. However, differences in VE by clade may not explain the potential birth cohort effect, as the VE estimates against A(H1N1)pdm09 clade 5a.2a were higher overall than those against 5a.2a.1, but also low for those aged 18–64 years (28%). In the analysis of A(H1N1)pdm09 VE by birth cohort we observed a signal of lower VE among those born 1976 to 1984. The first exposure of this population group to the influenza virus was the A(H1N1) A/USSR/90/77 or possibly A/Chile/1/83/CH83 (1976–1984). In the US, researchers also observed a birth cohort effect in their 2023/24 end-of-season results, with VE at 21% (95% CI: −6 to 41) among those aged 18–49 years [27]. However, in contrast to our study, they observed a lower VE among those aged 50–64 years as well at −8% (95% CI: −62 to 28). Sample size was too low to robustly estimate VE by year of birth by A(H1N1)pdm09 clade, which would help disentangle possible separate birth cohort and clade-specific effects. An alternative explanation could be that the patients in the 1976 to 1984 birth cohort were at greater risk of infection, perhaps due to a higher proportion of comorbidities. A descriptive analysis of comorbidities by birth cohort group, appended in Supplementary Table S4, did not support this, although factors other than comorbidities may play a role.

Antigenic results indicated that egg- and cell-based vaccine viruses recognised both circulating viruses well [24]. However, if only a narrow age group (e.g. born 1976 to 1984) is affected by a lower VE, then this may not be reflected in overall antigenic results. In addition, antigenic analyses are most often carried out in ferret sera, and antigenicity may differ between human and other species [29,30]. Different VE by birth cohort can be driven by one or more amino acid changes at specific positions in the influenza haemagglutinin surface protein between first and current influenza exposure [9,31,32]. At time of writing, we did not identify a specific amino acid mutation or mutations at a key position in circulating A(H1N1)pdm09 viruses compared with the A/USSR/90/77 and A/Chile/1/83 strains that could explain our results. We note that despite overall large sample size, VE by year of age and VE by clade had low precision, and random variation could play a role.

The VE against influenza A(H3N2) was 35% among all ages, ranging 34–43% by age group and among those in the target group for influenza vaccination. As few people ≥ 65 years in our study were infected with influenza A(H3N2), sample size did not allow us to estimate the VE for this age group. Our results are comparable with those from the previous seasons: in 2022/23 the VE was 36%, ranging 30–52% by age group and among the influenza vaccination target group [16]. The all-age 2023/24 interim influenza A(H3N2) VE at primary care level was 40% (95% CI: 5–61) in Canada, higher at 54% (95% CI: 11–77) in the US among adults and at 49% (95% CI: 26–65) in the UK among those aged 18–64 years [25,26,33]. All viruses sequenced belonged to clade 2a.3a.1, whereas the vaccine virus A(H3N2) component belonged to clade 2a. The all-age VE against A(H3N2) clade 2a.3a.1 was 38%, similar to our overall all-age estimate of 35%. There was variable recognition of the egg- and cell-based A(H3N2) vaccine component antisera for circulating A(H3N2) 2a.3a.1 subclades, and the vaccine strain was updated to A(H3N2) clade 2a.3a.1 A/Thailand/8/2022 (H3N2)-like virus or A/Massachusetts/18/2022 (H3N2)-like virus for the 2024/25 northern hemisphere influenza vaccine [24,34].

Against influenza B/Victoria, the VE was high for all age groups, at 70–92%. This is comparable to the 2022/23 season, where VE ranged between 72% and 84%, depending on age group [16]. This high VE was also seen in the US, with a 2023/24 outpatient VE of 78% among adults and 89% among children [33]. There were almost no influenza B/Victoria cases this season among those 65 years and older (n = 2), so we did not estimate the VE in this age group. Circulating viruses were antigenically similar to the B/Austria/1359417/2021 vaccine virus.

Limitations include small sample size for VE against influenza A(H3N2) and B/Victoria among older adults. Furthermore, one of the study sites (Spain, national level) represented 58% of the data and was therefore given more weight in the analysis than the other study sites. While the ratio of cases to controls becomes very small at the end of the study period, excluding the last weeks of analysis does not change the results (VE differs by ≤3%; data not shown). Even if the number of sequenced viruses was sufficient to estimate clade-specific VE, a larger sample size would increase power and reliability of the VE estimates, and to enable more detailed birth-cohort-specific VE by influenza A(H1N1)pdm09 clade. In the birth cohort-specific analysis, patient-specific information on past infections and vaccination would be relevant for interpretation of results, as these can affect immune responses to currently circulating influenza strains [4,35]. Finally, this observational study may be subject to unmeasured confounding, despite adjustment for potential confounders.

## Conclusion

Overall, the 2023/24 end of season VE in the VEBIS multicentre study at primary care was 51%, which indicates around one in two vaccinated people being protected against medically attended symptomatic influenza infection at primary care level. The results showed a high VE against influenza B/Victoria, with comparatively lower VE against influenza A(H1N1)pdm09 and A(H3N2). The VE was higher among children, with point estimates exceeding 60% for influenza A(H1N1)pdm09 and B/Victoria, and at 40% against influenza A(H3N2). These findings underline that influenza vaccination is an effective way of preventing influenza morbidity. We investigated VE against A(H1N1)pdm09 by birth cohort and noted potential birth cohort effects, with lower VE among those people with first likely A(H1N1) influenza infection with A/USSR/90/77 or A/Chile/1/83 (1976–1984). More research into birth cohorts within the VEBIS primary care network is planned.

## Data Availability

Aggregate data are available from the corresponding author at reasonable request from the corresponding author. The 1,487 sequences generated in connection with this analysis have been submitted to GISAID.

## Supplementary Data

498000 Supplementary Material
