# Supplementary material for: Influenza vaccine effectiveness in Europe and the birth cohort effect against influenza A(H1N1)pdm09: VEBIS primary care multicentre study, 2023/24
Source: Euro Surveill. 2025 Jun 12;30(23):2500011. doi: 10.2807/1560-7917.ES.2025.30.23.2500011 (PMC12164280; doi:10.2807/1560-7917.ES.2025.30.23.2500011)
Supplement: Supplementary Material [file 25-00011_KISSLING_Supplement.pdf]

Supplementary information

This supplementary material is hosted by *Eurosurveillance* as supporting information alongside the article "Influenza vaccine effectiveness in Europe and the birth cohort effect against influenza A(H1N1)pdm09: VEBIS primary care multicentre study, 2023/24", on behalf of the authors, who remain responsible for the accuracy and appropriateness of the content. The same standards for ethics, copyright, attributions and permissions as for the article apply. Supplements are not edited by *Eurosurveillance* and the journal is not responsible for the maintenance of any links or email addresses provided therein.

Supplementary Table S1: 2023/24 national influenza vaccination campaign and recruitment information, by study site, VEBIS primary care study, September 2023 – June 2024

Study site	Start of 2023/24 national influenza vaccination campaign	Case definition used for recruitment of patients[a]	Age-related influenza vaccination recommendations
Croatia	24 Oct 2023	EU-ILI or EU-ARI	≥65y
France	17 Oct 2023	Sentinelles ARI	≥65y; 2–17y[b]
Germany	1 Aug 2023	ARI	≥60y
Hungary	1 Oct 2023	EU-ARI	≥60y
Ireland	2 Oct 2023	EU-ARI	≥65y; 2–17y
The Netherlands	7 Oct 2023	EU-ILI or EU-ARI	≥60y
Portugal	29 Sep 2023	EU-ARI	≥60y
Romania	2 Oct 2023	EU-ILI or EU-ARI	≥65y; 6m–18y[b]
Spain, national	25 Sep 2023	EU-ARI	≥60y; 6m–4y
Spain, Navarre region	16 Oct 2023	EU-ILI	≥60y; 6m–4y
Sweden	7 Nov 2023	EU-ARI	≥65y

ARI: acute respiratory infection; EU: European Union; ILI: influenza-like illness; m: months; VEBIS: Vaccine Effectiveness, Burden and Impact Studies; y: years

[a] EU-ARI: Sudden onset of symptoms and at least one of four respiratory symptoms (cough, sore throat, shortness of breath, coryza) and a clinician's judgement that the illness is due to an infection; EU-ILI: Sudden onset of symptoms and at least one of four systemic symptoms (fever or feverishness, malaise, headache, or myalgia) and at least one of three respiratory symptoms (cough, sore throat, or shortness of breath); Sentinelles ARI: Sudden onset of fever (or feverishness), and respiratory signs; The ARI case definition in Germany includes patients with at least one of the following four symptoms: fever, cough, coryza or sore throat.

[b] Recommended, but not fully reimbursed; not included in the "target group for influenza vaccination" analysis.

Supplementary Table S2: Age and birth cohort groupings for analysis of vaccine effectiveness against A(H1N1)pdm09, VEBIS primary care study, September 2023 – June 2024

**Age group (years)**	0–15	16–25	26–38	39–47	48–56	57–67	68–90
**Birth cohort**	2008–2023	1998–2007	1985–1997	1976–1984	1967-1975	1956-1966	1933-1955
**Influenza A subtype of first infection**	A(H1N1) or A(H3N2)				A(H3N2)	A(H2N2)	A(H1N1)
**First likely A(H1N1) antigenic group encounter**	CA09 and later clusters	NE99 / BR07	SI86 / TE91	US77 / CH83	US77 / CH83	US77 / CH83	Sparse data

BR07: A/Brisbane/59/2007; CA09: A/California/07/2009; CH83: A/Chile/1/83; NE99: A/New Caledonia/20/1999; SI86: A/Singapore/6/1986; TE91: A/Texas/36/1991; US77: A/USSR/90/77.

Supplementary Table S3: Number and proportion of cases and controls by study site and age group, VEBIS primary care study, September 2023 – June 2024

		Age group							
		Cases				Controls			
		0-17	18-64	65+	Total	0-17	18-64	65+	Total
DE	n	614	389	34	1037	1997	1649	223	3869
	%	39	13	9	21	26	12	6	15
ES	n	527	1224	190	1941	4429	8233	2795	15457
	%	33	41	48	39	57	61	73	62
FR	n	225	405	38	668	654	951	184	1789
	%	14	14	10	14	8	7	5	7
HR	n	17	40	1	58	12	99	15	126
	%	1	1	0	1	0	1	0	1
HU	n	0	66	11	77	0	509	107	616
	%	0	2	3	2	0	4	3	2
IE	n	128	379	39	546	410	1015	240	1665
	%	8	13	10	11	5	8	6	7
NA	n	7	116	20	143	19	207	64	290
	%	0	4	5	3	0	2	2	1
NL	n	44	221	45	310	183	450	156	789
	%	3	7	11	6	2	3	4	3
PT	n	6	74	10	90	19	162	38	219
	%	0	2	3	2	0	1	1	1
RO	n	3	19	0	22	5	53	2	60

		Age group							
		Cases				Controls			
		0-17	18-64	65+	Total	0-17	18-64	65+	Total
	%	0	1	0	0	0	0	0	0
SE	n	12	39	5	56	18	85	28	131
	%	1	1	1	1	0	1	1	1
Total	n	1583	2972	393	4948	7746	13413	3852	25011
	%	100	100	100	100	100	100	100	100

Supplementary Figure S1: Number of influenza patients by birth cohort age group and vaccination status, and proportion of vaccinated cases, for influenza test-negative controls, A(H1N1)pdm09, clade 5a.2a and clade 5a.2a.1 cases, VEBIS primary care study, September 2023 – June 2024

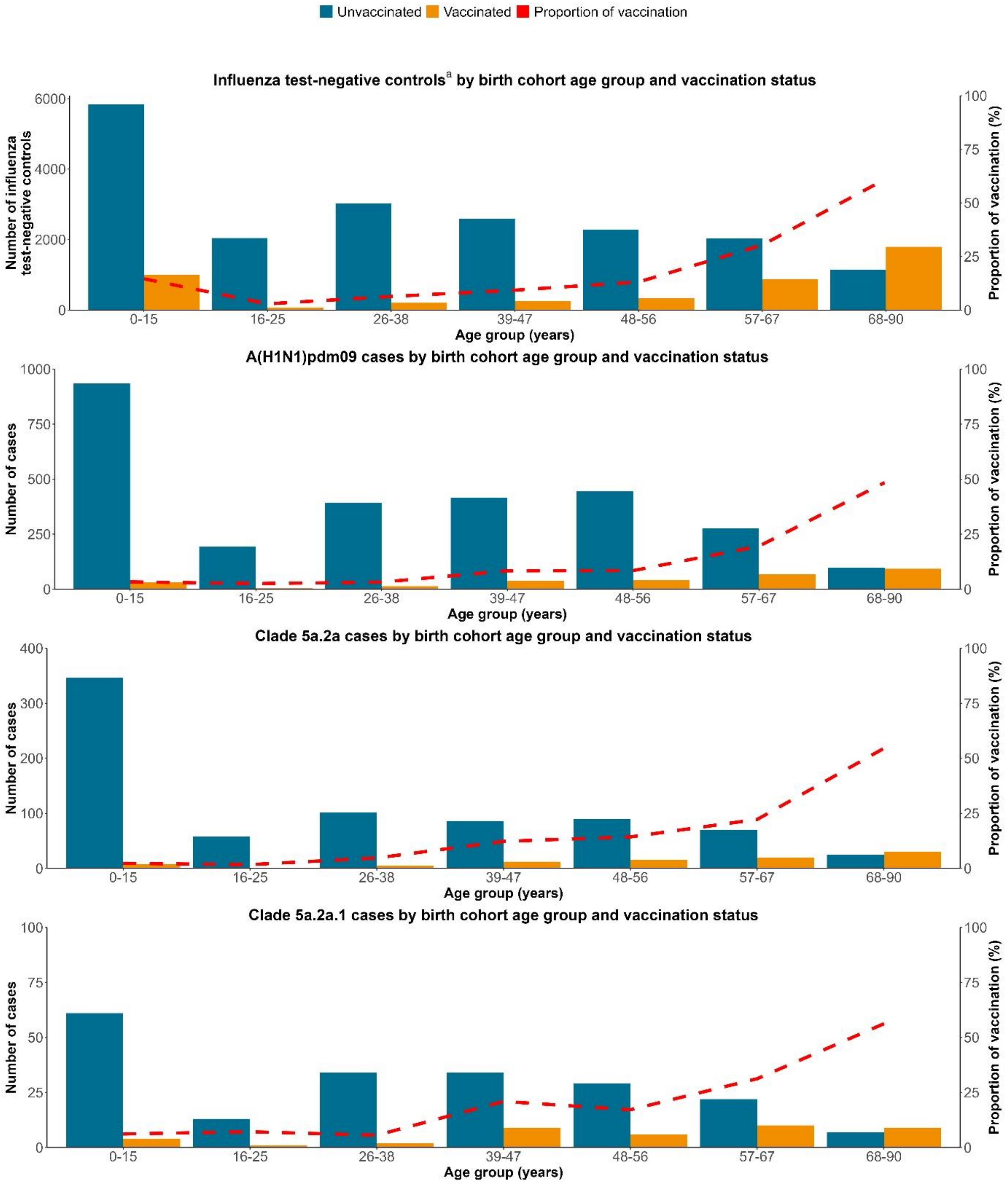

[a] Influenza test-negative controls correspond to the influenza A(H1N1)pdm09 analysis and controls with an onset week before or after an influenza A(H1N1)pdm09 case within each site are excluded.

Supplementary Figure S2: Vaccine effectiveness against any influenza, A(H1N1)pdm09, A(H3N2) and B, overall, by age group and among the target group for vaccination. VEBIS primary care multicentre study, September 2023 – June 2024

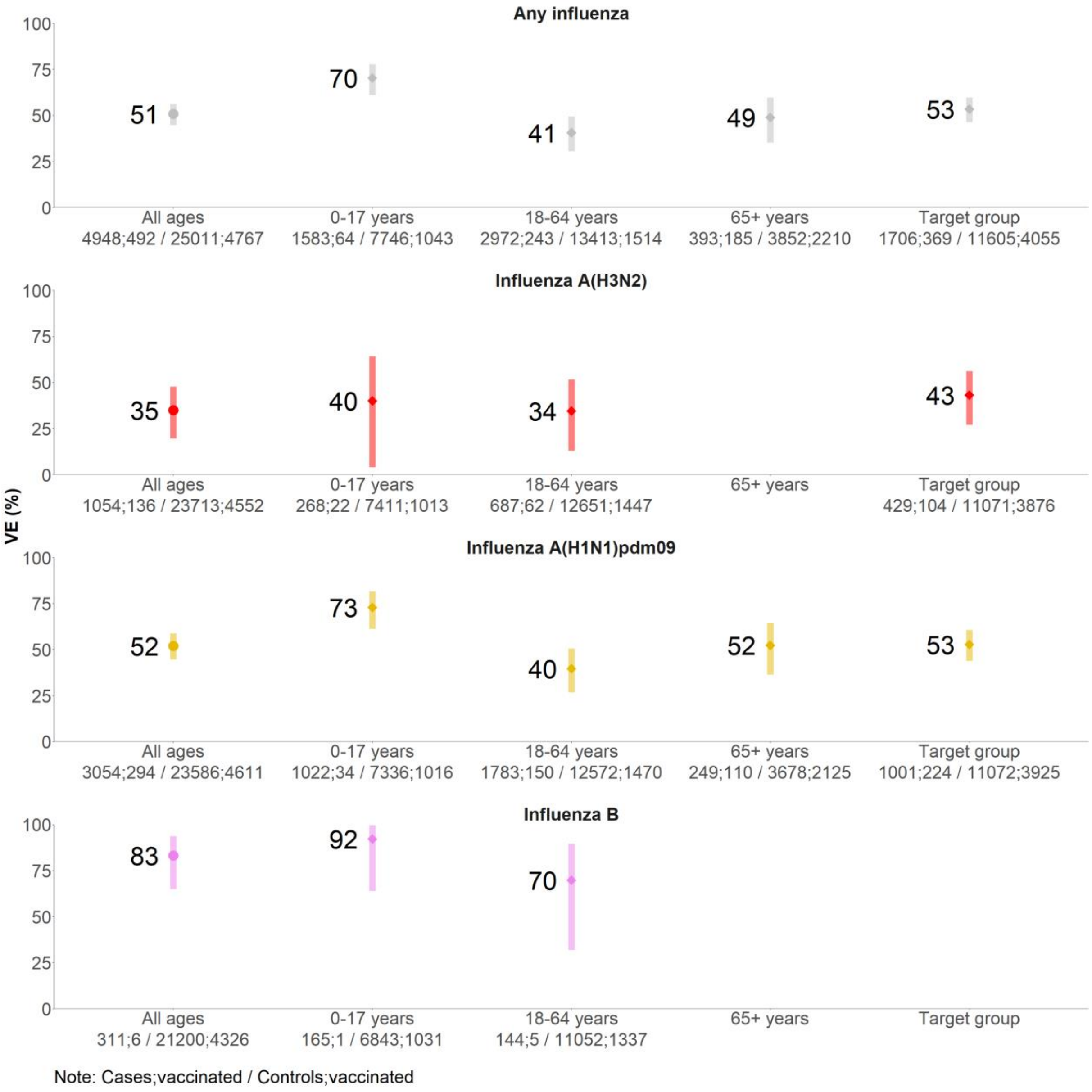

Supplementary Table S4: Number and proportion of A(H1N1)pdm09 cases and controls by presence of chronic conditions and birth cohort, VEBIS primary care study, September 2023 – June 2024

				Age group							
				0-15	16-25	26-38	39-47	48-56	57-67	68-90	Total
Presence of chronic conditions	Controls	Unvaccinated	n	348	219	336	471	618	863	786	3641
			%	**6**	**11**	**11**	**18**	**27**	**43**	**69**	**19**
		Vaccinated	n	155	19	60	92	179	483	1310	2298
			%	**16**	**31**	**29**	**35**	**52**	**55**	**73**	**51**
	Cases	Unvaccinated	n	55	18	46	45	98	102	65	429
			%	**6**	**9**	**12**	**11**	**22**	**37**	**66**	**16**
		Vaccinated	n	7	1	5	12	21	39	69	154
			%	**22**	**20**	**38**	**32**	**51**	**58**	**74**	**53**
